# Rewiring brain structural and functional disconnection with acupuncture in rat model of vascular cognitive impairment and dementia

**DOI:** 10.1186/s40659-026-00670-5

**Published:** 2026-01-19

**Authors:** Lu Wang, Ji-ping Zhao, Yan Cao, Si-Ming Ma, Jing-Wen Yang, Xin-Tong Su, Jin Huang, Qing-Yong Wang, Cun-Zhi Liu

**Affiliations:** 1 School of Acupuncture-Moxibustion and Tuina, Beijing University of Chinese Medicine, 11 Beisanhuan East Road, Chaoyang District, Beijing, 100029 China; 2 Dongzhimen Hospital, Beijing University of Chinese Medicine, 11 Beisanhuan East Road, Chaoyang District, Beijing, 100029 China

**Keywords:** Acupuncture, Connectional diaschisis, White matter lesions, Functional connectivity, Vascular cognitive impairment and dementia, Inflammatory

## Abstract

**Background:**

Connectional “diaschisis” theory indicates that the remote functional network disconnection resulting from focal white matter damage contributes to the cognitive dysfunction in vascular cognitive impairment and dementia (VCID). Acupuncture has been reported to attenuate white matter lesions and improve cognitive dysfunction of VCID individuals. However, whether these benefits are associated with the connectional diaschisis remains unclear.

**Methods:**

Here, 12-time acupuncture treatment was administered to the rat model of VCID. After behavioral tests, functional and diffusion magnetic resonance imaging were performed to analyze the connectivity of brain networks and white matter integrity. Pathological changes in myelin loss, axon injury, and glial activation were also observed. To clarify the interrelation of function and structure, correlation and mediation analyses were conducted.

**Results:**

Acupuncture ameliorated spatial working memory loss, reconnected the disruption of the default mode network, and prevented myelin and axon in the corpus callosum from the inflammatory attack. The association between restored corpus callosum integrity and spatial working memory was partially mediated by the increased functional connectivity of the anterior cingulate cortex.

**Conclusions:**

These evidence suggest that rewiring the corpus callosum-anterior cingulate cortex axis may be an integrated mechanism for the acupuncture effects on brain structure and function in VCID.

**Supplementary Information:**

The online version contains supplementary material available at 10.1186/s40659-026-00670-5.

## Introduction

White matter abnormalities (WMA) induced by chronic cerebral hypoperfusion are the essential features in vascular cognitive impairment and dementia (VCID) [1]. Patients with WMA develop variable cognitive deficits due to the diversity of affected subregions, mostly exhibit damaged executive function. WMA are represented by loss of myelin-associated protein, axonal degeneration, and altered diffusion tensor imaging (DTI) indices, which is initiated by hypoxia, but continuously progresses and deteriorates with the expansion of neuroinflammatory response. Focal structure lesions underlie the remote functional disconnection of brain networks, thus result in the cognitive impairment [2, 3]. The correlation between structure and function has been proved by a series of neuroimaging studies about connectional diaschisis, suggesting that the brain networks disruption, for example, default mode network (DMN) and central executive network, are the mediator that bridges the white matter injury and cognitive dysfunction of VCID individuals [4, 5].

Acupuncture is regarded as a promising alternative therapy that helps to regulate brain function [6, 7]. In neurological diseases such as Alzheimer’s disease and ischemic stroke, acupuncture promotes the functional connectivity and the efficiency of local networks [8]. In animal experiment, acupuncture protects the WMA of VCID rats by enhancing oligodendrocyte regeneration [9]. Although acupuncture can protect both brain structure and function, the underlying relationship remains unclear. In the current study, we combine imaging with pathology to explore whether acupuncture ameliorates spatial cognitive function of VCID rats by rewiring the structural and functional disconnection of white matter, thus providing a new insight into the treatment of VCID by acupuncture.

## Results

### Acupuncture attenuated cognitive impairment of VCID rats

**Fig. 1:**
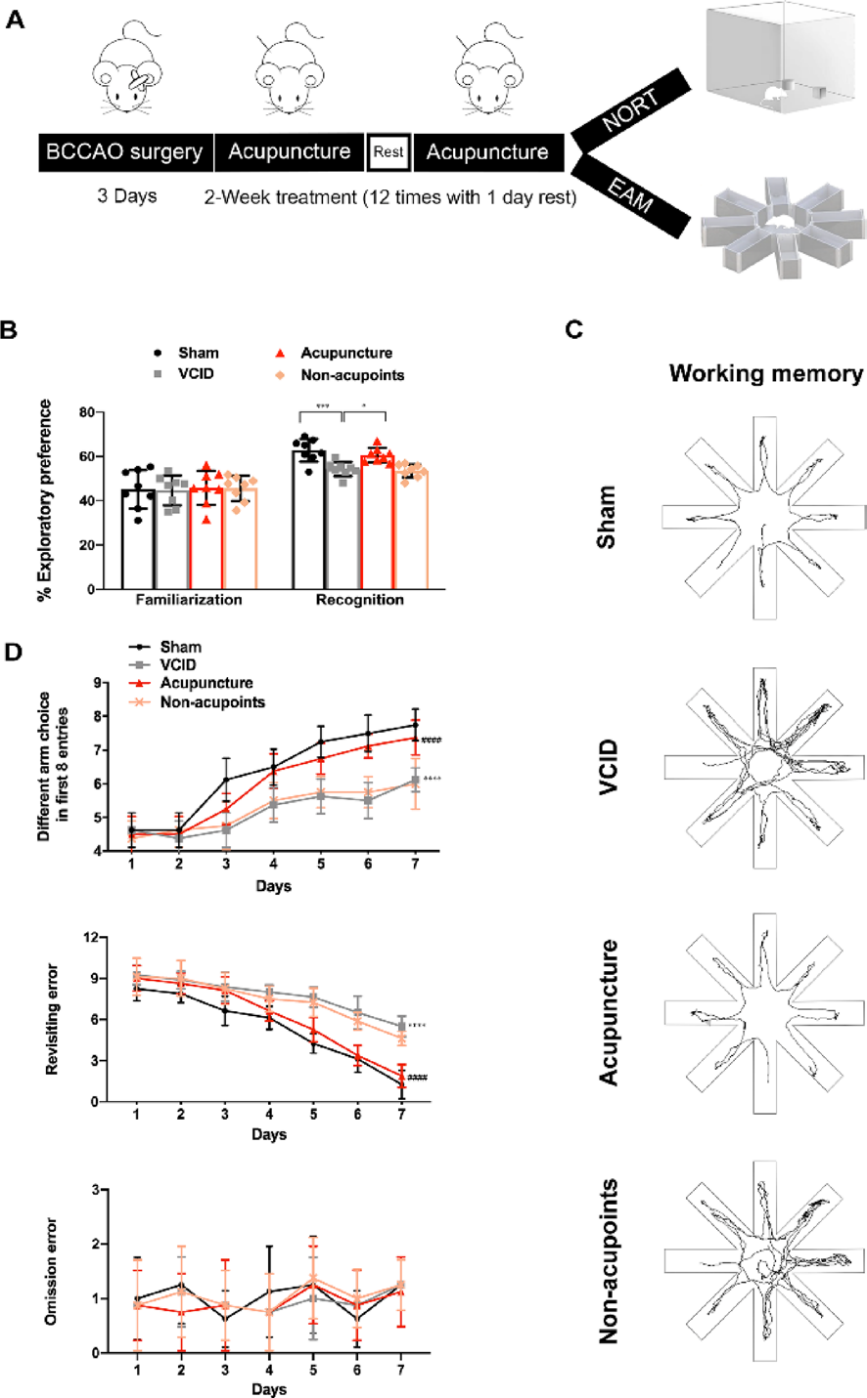
Acupuncture protected short-term and spatial working memory of VCID rats.** A** The experimental procedure. **B** The exploratory preference for the old and novel object of rats. One-way ANOVA with Tukey’s post-hoc test. Data are presented as mean ± SD (*n* = 8 per group). * *p* < 0.05 and *** *p* < 0.001, respectively. **C** Working memory of rats was evaluated by eight-arm maze during the training and probe trials after BCCAO surgery. **D** Presentative moving trace of each group in eight-arm maze probe trails of working memory. Two-way ANOVA with Tukey’s post-hoc test. Data are presented as mean ± SD (*n* = 8 per group). ****p < 0.001, compared with Sham group; #### *p* < 0.001, compared with VCID group. NORT, novel object recognition test; EAM, eight-arm maze; Sham, sham-operated group; VCID, BCCAO-operated group; Acupuncture, VCID + acupuncture at GV20 and ST36 group; Non-acupoints, VCID + acupuncture at non-acupoints group

First, we had the animals trained and probed in the novel object recognition test and eight-arm maze to evaluate the effects of acupuncture on memory performance (Fig. 1A). In the novel object recognition test, VCID rats exhibited less preference for exploring novel object during the recognition stage, while acupuncture promoted the recovery of this short-term memory (Fig. 1B). Next, in the eight-arm maze assessment of spatial working memory, a significant time effect (F [6, 168] =  107.6, *p* < 0.0001; F [6, 168]  =  239.1, *p* < 0.0001)), treatment effect (F [3, 28] = 51.95, *p* < 0.0001; F [3, 28] = 29.52, *p* < 0.0001), and the interaction of time and treatment (F [18, 168] = 4.897, *p* < 0.0001; F [18, 168] = 7.373, *p* < 0.0001) were found in the different arm choices in first 8 entries and revisiting error. Compared with the sham operation, bilateral common carotid artery occlusion (BCCAO) surgery resulted in fewer different arm choices in the first 8 entries and more revisiting errors in the probe trial (Fig. 1C and D), suggesting a working memory loss. After acupuncture, rats showed better performance in the working memory test. However, the time effect on omission error was significant (F [6, 168]  =  2.288, *p* < 0.0378), but no apparent treatment effect (F [3, 28] = 0.2076, *p* = 0.8903) or the interaction of time and treatment (F [18, 168] = 0.413, *p*  =  0.9838) was found in omission error, indicating that the motivation of feeding was not impaired (Table S1). Additionally, the beneficial effects of acupuncture on memory were not seen in the Non-acupoints group. These results confirm that acupuncture treatment ameliorates the short-term and spatial working memory of VCID rats. Besides, the open-field test showed that the distinct differences in total distance and walking speed were not found among groups, implying that BCCAO surgery and acupuncture did not impair the animal’s motor ability (Fig. S1).

### Acupuncture modulated remote cortical network of VCID rats

**Fig. 2:**
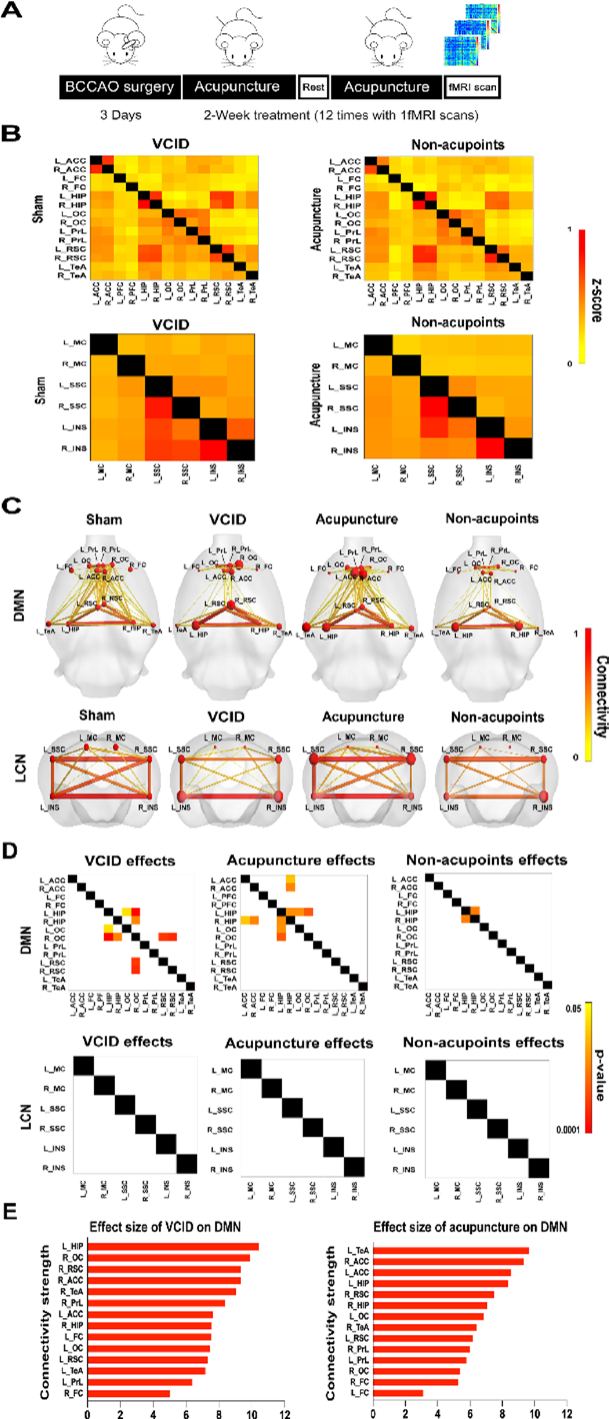
Acupuncture modulated the function of DMN in VCID rats.** A** Schematic of the fMRI scanning design. **B** Group-averaged DMN and LCN functional connectivity (z-score) matrices for sham, VCID, Acupuncture and Non-acupoints rats. Left bottom matrices show the Sham and Acupuncture groups, right top matrices show the VCID and Non-acupoints groups. Functional connectivity is colored according to the z-score. **C** Visualizing diagrams demonstrate the functional connectivity strength (edge) changes between brain regions (node) in DMN and LCN. **D** Adjusted *p* value matrices highlight the significant changes of functional connectivity between Sham and VCID, VCID and Acupuncture, and VCID and Non-acupoints group (*p* < 0.05, corrected with FDR). **E** The sum of effect sizes (Cohen’s *d*) across all connections in DMN was calculated to show which brain regions are more affected by BCCAO surgery or acupuncture treatment. Data are presented as mean ± SD (*n* = 8 per group). DMN, default mode network; LCN, lateral cortical network; FDR, false discovery rate; Sham, sham-operated group; VCID, BCCAO-operated group; Acupuncture, VCID + acupuncture at GV20 and ST36 group; Non-acupoints, VCID + acupuncture at non-acupoints group; L, left; R, right; ACC, anterior cingulate cortex; FC, frontal cortex; HIP, hippocampus; OC, orbital cortex; PrL, prelimbic cortex; RSC, retrosplenial cortex; TeA, temporal association cortex; MC, motor cortex; SSC, somatosensory cortex; INS, insula cortex

Given that acupuncture protected memory and that the brain network helps to demonstrate specific changes in brain function, we performed partial analyses within DMN and lateral cortical network (LCN) (Fig. 2A), which were reported as two discriminate local networks that are related to working memory. DMN matrices and visualizing results suggest that in the VCID group, functional connectivity between the right orbital cortex and the bilateral hippocampus, the left orbital cortex and the left hippocampus, and the right orbital cortex and the bilateral retrosplenial cortex were significantly reduced as compared with Sham group. In contrast, the reduced functional connectivity between the bilateral orbital cortices and the left hippocampus was reversed by acupuncture at GV20 and ST36 (Fig. 2B-D). Interestingly, acupuncture also improved functional connectivity that was not damaged by BCCAO surgery, including the one between the right hippocampus and the right anterior cingulate cortex and one between the left hippocampus and the right hippocampus, which may be related to inhibition of hippocampal oxidative stress (Fig. S2). For LCN, any substantially decreased functional connectivity (Fig. 2 B-D) was not observed between brain regions. Unexpectedly, acupuncture at non-acupoints showed minor effects on DMN, appeared as increased functional connectivity between the bilateral hippocampus (Fig. 2B-D). These results indicate that the improvement of acupuncture on cognitive dysfunction of VCID rats is manifested as the enhancement of specific functional connectivity in DMN.

To quantify the effect size of affected functional connectivity in VCID and Acupuncture groups, we calculated the connectivity strength (sum of Cohen’s *d*) of regions of interest (ROIs) at the level of local networks (Table S2). In DMN, connectivity strength of the left hippocampus, right orbital cortex and left retrosplenial cortex was more vulnerable to the VCID, while the left temporal association cortex and bilateral anterior cingulate cortices were more affected by acupuncture (Fig. 2E).

### Acupuncture improved subcortical white matter integrity of corpus callosum

**Fig. 3:**
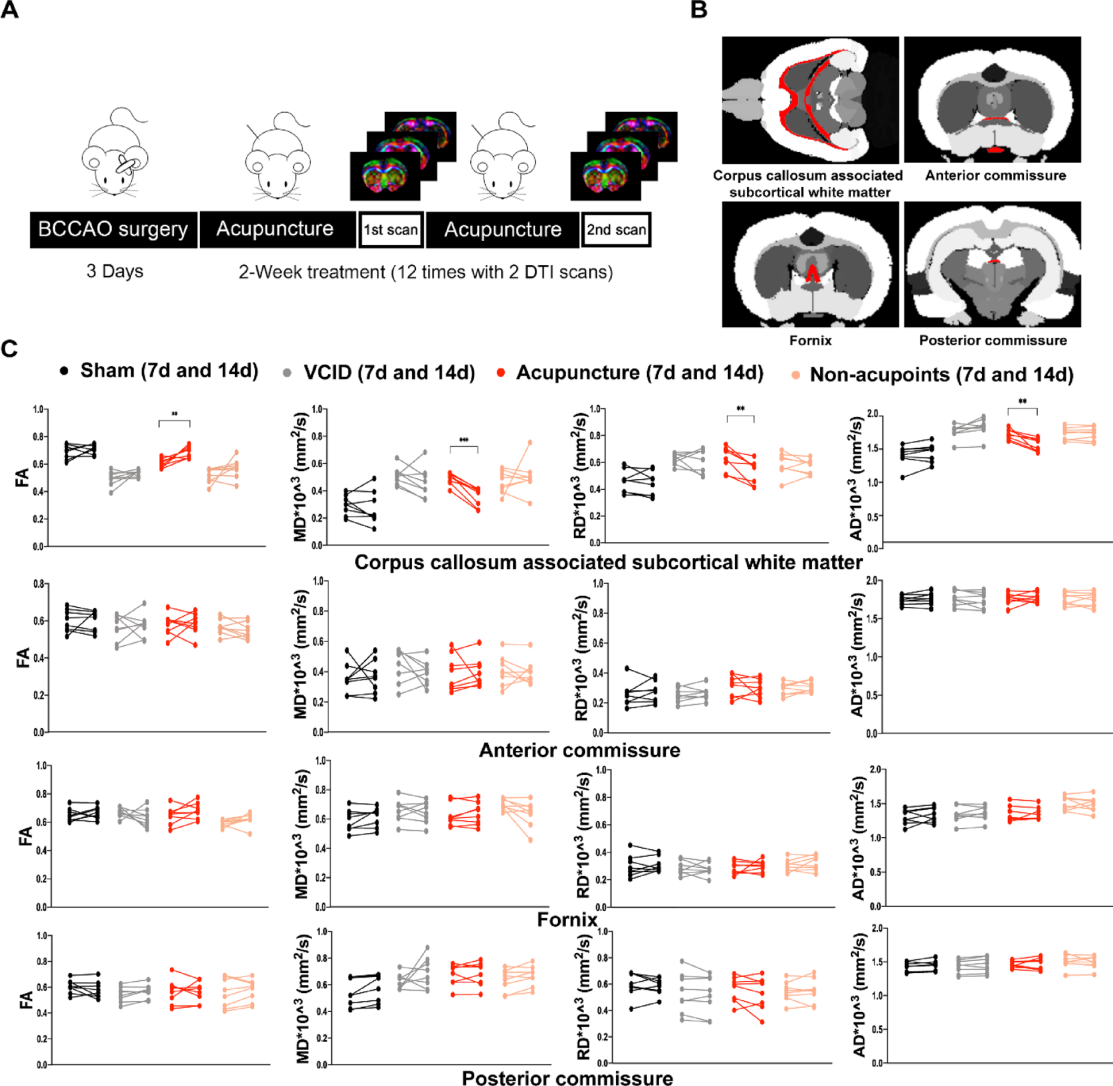
Acupuncture improved white matter integrity of VCID rats.** A** Schematic of the DTI scanning design. **B** The white matter ROIs drawn from rat brain template. **C** Diffusion tensor imaging indices in ROIs including FA, MD, RD and AD were quantified at 7 days and 14 days after BCCAO surgery. Paired* t*-test. Values are presented as mean ± SD (*n* = 8 per group). ** *p* < 0.01, *** *p* < 0.001, respectively. FA, fractional anisotropy; MD, mean diffusivity; RD, radial diffusivity; AD, axial diffusivity; Sham, sham-operated group; VCID, BCCAO-operated group; Acupuncture, VCID + acupuncture at GV20 and ST36 group; Non-acupoints, VCID + acupuncture at non-acupoints group; ROIs, regions of interest

In view of the essential role of WMA in the pathogenesis of VCID, we further probed whether acupuncture could promote white matter integrity. DTI can reflect the diffusion of water molecules and the integrity of white matter fiber bundles [10–12]. We calculated DTI indices of corpus callosum and associated subcortical white matter (CCWM), anterior commissure (AC), fornix (FN), and posterior commissure (PC) (Fig. 3A) on the 7th and 14th days after BCCAO surgery because these white matter ROIs have been proved to be correlated with cognitive function [3, 13, 14]. (Fig. 3B) The intra-group comparison suggested that fractional anisotropy, mean diffusivity, radial diffusivity and axial diffusivity in CCWM were remarkably improved, indicating that myelin and axon were both rescued by a 2-week acupuncture treatment (Fig. 3C). Furthermore, inter-group comparison 14 days after surgery was also conducted to evaluate the deterioration or improvement of white matter integrity in different brain regions (Fig. S3). Among the representative ROIs, CCWM was both disease- and acupuncture-specific white matter region.

**Fig. 4:**
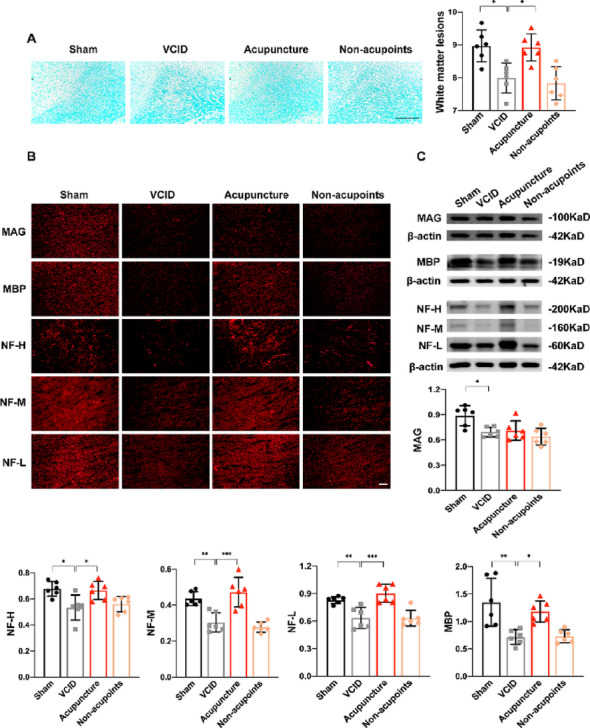
Acupuncture attenuated myelin loss and axonal degeneration within corpus callosum.** A** White matter organization was evaluated by LFB staining. Scale bar: 200 μm. One-way ANOVA with Tukey’s post-hoc test. Data are presented as mean ± SD (*n* = 6 per group). * *p* < 0.05. **B** The immunofluorescent expression of myelin associated proteins MAG and MBP, and axon structural proteins NF-H, -M, -L, Scale bar: 100 μm (*n* = 6 per group). **C** The quantification of MAG, MBP, NF-H, -M, -L were performed as the histograms. One-way ANOVA with Tukey’s post-hoc test. Data are presented as mean ± SD (*n* = 6 per group). * *p* < 0.05, ** *p* < 0.01, *** *p* < 0.001, respectively. LFB, Luxol fast blue; MAG, myelin associated glycoprotein; NF, neurofilament; Sham, sham-operated group; VCID, BCCAO-operated group; Acupuncture, VCID + acupuncture at GV20 and ST36 group; Non-acupoints, VCID + acupuncture at non-acupoints group

To extend findings related to white matter integrity, we evaluated the pathology of corpus callosum in great detail. As shown by Luxol fast blue (LFB) staining, white matter organization was sparse in the VCID group, which was preserved by acupuncture but not non-acupoints (Fig. 4A). Since myelin sheath and axon are a basic component of white matter, we detected and quantified the expression of their associated proteins. We found that MBP and MAG, the markers for myelin, were remarkably decreased in the corpus callosum of VCID rats, whereas acupuncture only increased the expression of MBP but not MAG (Fig. 4B-C). We also investigated the expression of NF, a marker for axon integrity, and found that three subunits of NF, including NF-L, NF-M, and NF-H were all compromised in VCID group (Fig. 4B–C). On the contrary, their expression in corpus callosum was significantly increased after acupuncture treatment (Fig. 4B–C). The above results demonstrate that acupuncture protected myelin and axon integrity of corpus callosum in VCID rats.

### Acupuncture improved white matter integrity by regulating microglia polarization within the corpus callosum

**Fig. 5:**
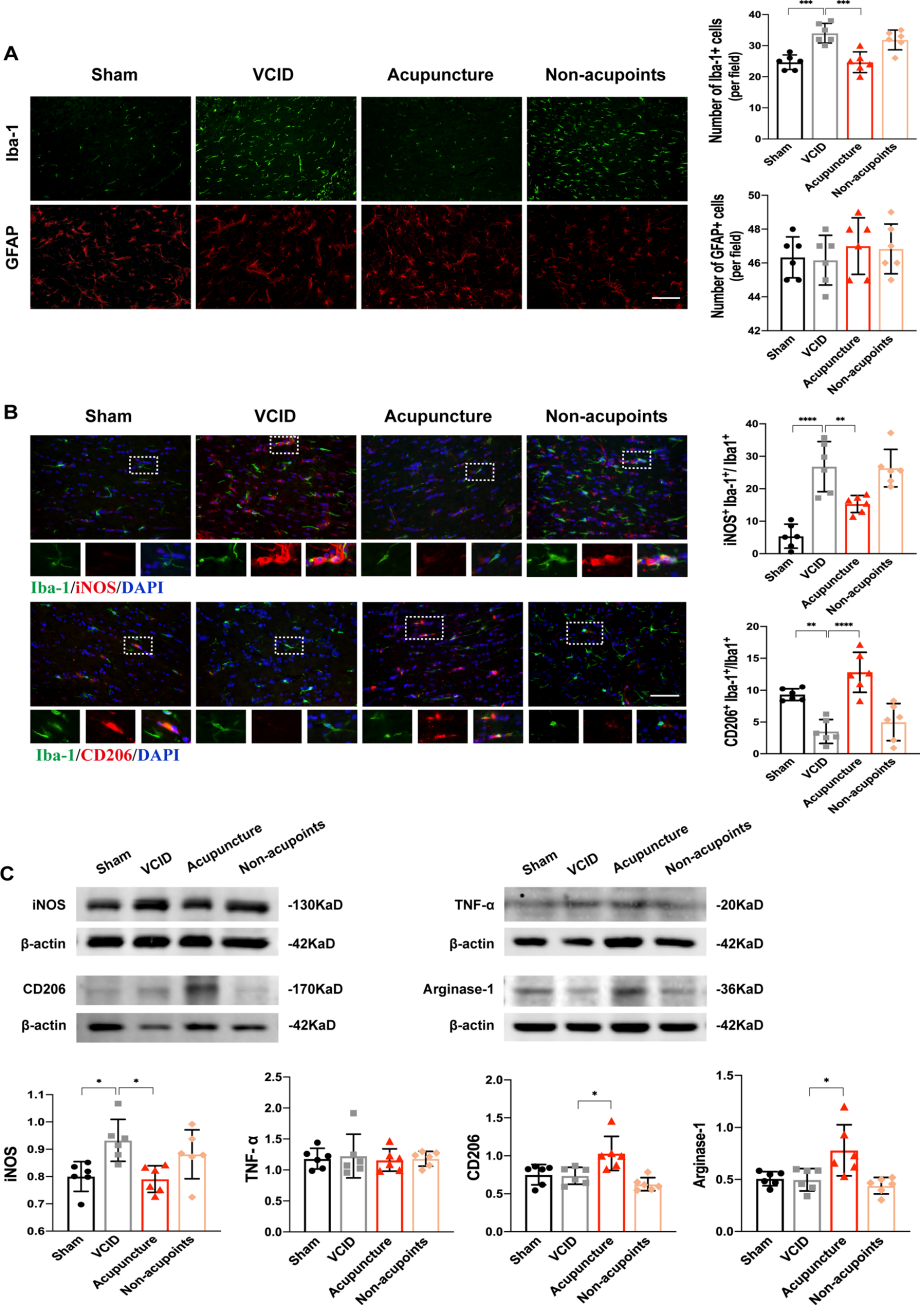
Acupuncture inhibited white matter inflammation induced by microglia polarization.** A** Representative images demonstrate the immunofluorescent labeling of Iba-1^+^ cells and GFAP^+^ cells, Scale bar: 100 μm. One-way ANOVA with Tukey’s post-hoc test. Data are presented as mean ± SD (*n* = 6 per group). *** *p* < 0.001. **B** The colocalization of iNOS and Iba-1, and CD206 and Iba-1 suggest the phenotypes of microglia polarization in the VCID and Acupuncture group, Scale bar: 100 μm. One-way ANOVA with Tukey’s post-hoc test. Data are presented as mean ± SD (*n* = 6 per group). ** *p* < 0.01, **** *p* < 0.0001, respectively. **C** The protein expression of pro-inflammatory markers iNOS and TNF-α, anti-inflammatory markers CD206 and Arginase-1 were detected by Western blot. Quantitative analyses are shown in histograms. One-way ANOVA with Tukey’s post-hoc test. Data are presented as mean ± SD (*n* = 6 per group). * *p* < 0.05. Iba-1: ionized calcium binding adapter molecule 1, GFAP: glial fibrillary acidic protein, iNOS: inducible nitric oxide synthase, Sham: sham-operated group, VCID: BCCAO-operated group, Acupuncture: VCID + acupuncture at GV20 and ST36 group, Non-acupoints: VCID + acupuncture at non-acupoints group

It is well established that glial activation induced neuroinflammation is the main contributor to WMA in VCID models [15]. Our previous study showed that the levels of IL-1β and IL-6 were elevated in BCCAO group. Acupuncture markedly attenuated the levels of IL-1β and IL-6 in corpus callosum of BCCAO rats [16]. Therefore, in the current study, we further detected the expression of Iba-1 and GFAP, markers for microglia and astrocyte respectively, in the corpus callosum (Fig. 5A). We found a significantly increased number of Iba-1^+^ cells in the VCID and Non-acupoints groups compared to the Sham group, while acupuncture inhibited Iba-1 expression induced by BCCAO surgery. Although GFAP^+^ cells tended to have a greater expression in VCID rats, it showed no difference between groups (Fig. 5A).

Except accumulation, microglia activation and cytokines release keep white matter in a pro-inflammatory environment, thus resulting in WMA. Then we explored whether acupuncture affects the process of microglia polarization. Compared to the Sham group, chronic ischemia significantly increased the percentage of iNOS^+^/Iba-1^+^ microglia in the VCID group (Fig. 5B). Similarly, a trend toward a greater increase of pro-inflammatory cytokines (iNOS) in the corpus callosum was found, while anti-inflammatory markers (CD206 and Arginase-1) could hardly be detected in VCID rats (Fig. 5B). Acupuncture at true acupoints but not non-acupoints switched microglia polarizing to anti-inflammatory phenotype from pro-inflammatory phenotype, accompanied by a rebalance of pro-inflammatory and anti-inflammatory cytokines levels (Fig. 5C). These evidence suggest that acupuncture protects white matter integrity by regulating the balance of microglia polarization in the corpus callosum.

### Acupuncture ameliorated cognitive dysfunction via rewiring connectional diaschisis of corpus callosum-anterior cingulate cortex axis

The above results indicate that brain structural and functional disconnection were restored by acupuncture in VCID rats. To find out their roles in cognitive function after acupuncture treatment, we had a *post hoc* correlation analysis between the DTI indices of the corpus callosum and working memory. We found that fractional anisotropy, mean diffusivity, and axial diffusivity value of CCWM were all negative correlated with revisiting error (Table S3). Using a similar paradigm, we continuously analyzed the relationship between altered functional connectivity in DMN and cognitive function. There was a significant negative correlation between several functional reconnection and fewer revisiting errors after acupuncture treatment (Table S4). Compared with intra-hemispheric functional connectivity, according to the significance of the *p* value, inter-hemispheric functional reconnection was more closely related to the improvement of working memory (Table S4).

**Fig. 6:**
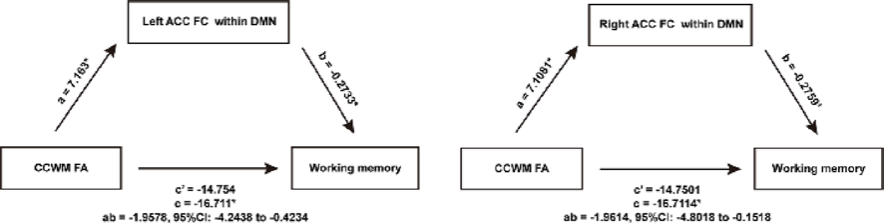
Acupuncture ameliorated cognitive dysfunction through rewiring the diaschisis of brain white matter structure and functional networks. *ab* is the indirect effect; path *c* represents the total effect; path *c’* represents the direct effect. The values of *a*,* b*,* c*, and *c’* are represented by regression coefficients (unstandardized). *p < 0.05. ACC, anterior cingulate cortex; CCWM, corpus callosum associated subcortical white matter; DMN, default mode network; FA, fractional anisotropy; FC, functional connectivity

In view of the apparent contribution of white matter integrity and functional connectivity to the cognitive function, we conducted a mediation analysis limited to the brain regions (orbital cortex, hippocampus and anterior cingulate cortex) that significantly correlate with working memory in Table S4, which helps to explain the relationship between white matter structure, DMN and cognitive performance after acupuncture. We found that functional connectivity of left and right anterior cingulate cortex in DMN mediated the association between fractional anisotropy of CCWM and revisiting errors, accounting for 11.72% and 11.74% of the total effect, respectively (Fig. 6). Besides, pathological examination confirmed that acupuncture enhanced the projections from the corpus callosum to the anterior cingulate cortex (Fig. S4). Together with the effect size data from Fig. 2E, these results indicate that acupuncture modulates specific functional connectivity by restoring focal white matter integrity, thus promoting recovery of working memory.

## Discussion

This study investigated the rewiring mechanism of acupuncture on brain connectional diaschisis in animal model of VCID. According to the current results, there may be a structure-function axis that underlies the maintenance of cognitive function. Acupuncture pulls the trigger at subcortical structural injury but induces the functional response of cortex. This process could be characterized as the association between corpus callosum integrity and anterior cingulate cortex functional connectivity in both disease and treatment states. The corpus callosum-anterior cingulate cortex axis may be regarded as a potential hallmark that partially mediates the acupuncture effects in VCID.

White matter is responsible for information processing and is likely more vulnerable to global ischemia than gray matter due to the watershed pattern of cerebral blood flow supplying it [17]. Previous studies have confirmed that in VCID patients, almost all the white matter regions appeared as altered DTI indices where the genu of corpus callosum exhibited the most injured integrity, and the decreased fractional anisotropy and increased mean diffusivity are the most frequently reported [18–20]. For the animal model of VCID, the intensive changes of axial diffusivity and radial diffusivity in cognition related brain regions were also recorded [13, 21]. Our experiment shows that the breakdown of overall organization and microstructure could be restored by acupuncture treatment, which is consistent with those published data. Considering rigorousness, we observed histological changes and verified that the protective effect of acupuncture was due to the attenuation of demyelination and axonal degeneration. Here, the convergence of imaging and pathological results might provide a wide range of evidences for acupuncture effects in VCID.

The process of glia activated to a neuroinflammatory type by phagocytosis of myelin debris with the hypoperfusion persist and progressive myelin breakdown is described as pattern recognition receptors mediated damage-associated molecular patterns, by which contributes to the form of pro-inflammatory environment [22]. Several studies have underpinned anti-inflammatory effects of acupuncture on brain tissue via targeting at microglia dysfunction in neurodegenerative diseases [23, 24]. A recent study found that acupuncture at multi-acupoint relieves proinflammatory microglia accumulation by inhibiting the signaling transduction of the RhoA/ROCK pathway in traumatic brain injury [24]. These outcomes prompted us to have a proposal that acupuncture might restore cerebral homeostasis through inhibiting microglial polarization. Although the changes in downstream pathway were not elaborated in this study, we pathologically proved that acupuncture regulates imbalance between proinflammatory and anti-inflammatory phenotypes of microglia. Acupuncture seems to be a regulator between pro-inflammation and anti-inflammation.

DMN and LCN are a pair of contradictory networks that can be activated when the brain is not engaging in a specific task or in an executive task requiring attention [25]. Rewiring of DMN abnormal networks is recognized as hallmarks of acupuncture treatment in patients with Alzheimer’s disease [26]. It has been proposed that rat possesses an intrinsic network that is similar to human DMN, in which temporal-prefrontal subsystem (e.g. cingulate cortex, orbital cortex, prelimbic cortex, temporal association cortex), parietal subsystem (e.g. retrosplenial cortex, hippocampus, parietal cortex) and their interaction are involved in the information processing of learning and memory [27]. In our study, attenuated functional connectivity of the hippocampus, orbital cortex and retrosplenial cortex (a homologous region of human posterior cingulate cortex) suggest dysfunction of these DMN subsystems. Moreover, these three regions constitute a complex feedback circuit that controls episodic memory. The functional disconnection between them may, at least in part, explain the behavioral changes seen in rat model of VCID. For the effects of acupuncture treatment, it warrants mentioning that the anterior cingulate cortex and temporal association cortex serve as the predominant nodes. As the specific component of the rat DMN, they are involved in visual processing and rewarding learning [28]. In the physiological state, its activation enhances the integration of multimodal sensory activity which helps to guide behavior [29]. We speculate that acupuncture effects on the anterior cingulate cortex and temporal association cortex along with improved function of hippocampus, may be a compensation for improving cognitive impairment of VCID rats.

The theory that WMA primarily results in desynchronization in linked long-range brain networks has been widely accepted as the “connectional diaschisis” reason for cognitive dysfunction [3, 30, 31]. Several convincing studies about vascular cognitive dysfunction demonstrate a mediation role of the posterior cingulate cortex between cingulum and global cognitive impairment [3]. This discovery reinforces the idea that disconnected brain networks bridge the WML and cognitive dysfunction. Consistent with the above hypothesis, our mediation analysis indicates that functional connectivity of anterior cingulate cortices is intermediation partially connecting corpus callosum lesions and working memory error in rats. This observation indicates that cingulate-retrosplenial cortices as the central part in integrative function of rodent DMN [25, 32], are sensitive to the hypoperfusion and acupuncture stimulation in VCID [33]. Accordingly, our findings highlight that corpus callosum-anterior cingulate cortex axis is not only likely to be a hallmark explaining the mechanism of acupuncture in the animal model of VCID, but provides a translational window by which other advanced non-pharmaceutical interventions may effectively improve cognitive symptoms in VCID patients.

Some technical and design defects in this study including thicker slice thickness (fMRI: 1 mm, DTI: 0.8 mm), relatively coarse imaging resolution and imprecise brain map limit our ability to distinguish the subdivision of several brain regions. For example, the inferior parietal lobe from the posterior parietal lobe, or genu from corpus callosum. Due to these limitations, we failed to determine the specificity of some cortical and subcortical subregions. Another limitation is that the baseline of DTI indices was not measured. Although the species, gender, age and weight of rats are strictly controlled, the individual difference may cause statistical bias.

## Conclusions

Overall, we macroscopically and microscopically investigated the rewiring effects of acupuncture on brain structure and function. Our results reveal that acupuncture improves cognitive performance through rewiring the disconnection between white matter and DMN. Among the affected brain regions, the corpus callosum-anterior cingulate cortex axis may be potential key component, reflecting the regulating pattern of acupuncture on connectional diaschisis in VCID.

## Materials and methods

### Animals and groups

A total of 120 Wistar rats (male, 8-week-old, Vital River Laboratory Animal Technology Co. Ltd, Beijing, China) weighing 300–320 g were used in this study. The animals were housed in groups with a controlled temperature (24 ± 0.5 ℃) on cycles of 12 h light/12 h dark with food and water available *ad libitum*. All rats were randomly divided into a sham-operated (Sham) group and a model group (Fig. S5). In the model group, rats were randomized into a bilateral common carotid artery occlusion (VCID) group, a VCID treated with acupuncture (Acupuncture) group, and a VCID treated with non-acupoints (Non-acupoints) group.

### Surgery procedure of BCCAO

VCID model was established by BCCAO surgery as previously described [34–38]. In summary, rats were anesthetized with an intraperitoneal injection of sodium pentobarbital (40 mg/kg) and maintained on a heating pad (37 ℃) during the surgery. 2% lidocaine (0.1mL) was injected intramuscularly to relieve the pain. The common carotid arteries were exposed and double ligated with 4− 0 silk sutures after a mid-cervical incision was made. In the Sham group, the same operation was performed as in the VCID group except for artery occlusion. The blind rats that exhibited significant weight loss and disability to finish the behavioral tests were removed from their group to guarantee the accuracy of experiment outcomes.

### Acupuncture treatment

3 days after the operation, awake rats received acupuncture treatment at “Baihui” (GV20) and bilateral “Zusanli” (ST36), or at non-acupoints (Table S5), once daily for 14 days, with one-day rest after six days [34, 39]. The one-day rest after six treatments was supposed to be consistent with the clinical practice, where outpatients usually take regular rest weekly during therapy. Acupuncture needles (Hwato, China, 0.22 × 5 mm) were used and retained in GV20 and bilateral ST36 synchronously in the Acupuncture group, or retained in bilateral non-acupoints synchronously in the Non-acupoints group for 15 min. The rats in the Sham and VCID groups underwent the same catching-grasping stimulation without the acupuncture.

### Novel object recognition test

Short-term memory was detected by the novel objective recognition test as the previous instruction within 24 h after the acupuncture intervention was finished. (i) habituation: The rats were placed in a testing box (40 cm × 40 cm × 40 cm) to the habituate to the environment for 30 min each day for 2 consecutive days. (ii) familiarization: On day 3, each rat was placed between two objects in the arena and allowed to explore for 5 min. After the exploration, rats would be sent to their home cages and had 1 h rest. (iii) recognition: After rest, one object was replaced by a new one with a similar size but different shape and color. Rats were allowed to explore the objects again for 2 min. The center of the rat’s head oriented within 45◦ and 4 cm of the novel object would be regarded as one novel object exploration. Leaning against, climbing over, or sitting on an object was not a valid exploration. The objects and box were cleaned with ethanol after each test to diminish the influence of olfactory traces. The percentage exploratory preference was calculated: time exploring novel object/ (time exploring old object + time exploring novel object) × 100% [40].

### Eight-arm maze

Spatial learning and memory were assessed by the eight-arm maze as described [41] 5 days after acupuncture treatment. (i) adaptive training: several salient cues were used for spatial orientation. Rats were partially deprived of food to maintain 80% of their body weight (Table S1), and allowed to retrieve food pellets scattered over the whole maze for 5 min (2 training per day, 4 training). (ii) working memory test: food pellets were placed at the end of all eight arms. Each rat was placed on the central platform of the maze in the begthe frame of apparatus above the maze to record the trials. Video tracking software (Jiliang software technology, CN) was used for quantification of behavior performance.

Inning to retrieve all food pellets for 10 min or until they finishded the task. Reentry into arms that had been visited was defined as working memory error. Visit an arm without food consumption, as an indication of motivation, was regarded as an omission error. For each trial, different arm choices in the first eight entries, revisiting error, and omission error were calculated. A video camera was fixed on.

### Open field test

An open field area (100 cm × 100 cm × 30 cm) made of black PVC was used to assess spontaneous activity. After acclimation to the testing room for 2 h, mice were tested for 10 min in the open field area. A video-tracking system (Jiliang software technology, CN) was used to measure the spontaneous activity of the animal. All traveled distances and the walking speed were recorded and analyzed [42].

### MRI scanning

MRI was acquired with a 7.0 T MR scanner (Bruker, GER) during or after acupuncture therapy (Fig. 1A). To obtain eligible images, rats were induced anesthesia with 2% isoflurane before scanning and maintained with 1.8% isoflurane (70 cycles/min) during acquisition. Respiration and blood oxygen saturation were monitored during scanning.

MRI acquisition: Rapid acquisition with relaxation enhancement was used as sequence of T_2_WI with the following parameters: TR = 5000 ms; TE = 36 ms; flip angle = 180°; slice thickness = 0.7 mm; matrix = 256 × 256; field of view = 35 × 35 mm.

DTI acquisition: 32 slices were captured with spin echo planar imaging sequence. TR = 6250 ms; TE = 22 ms; flip angle = 90°; slice thickness = 0.8 mm; matrix = 128 × 128; field of view = 30 × 30 mm; *b* values = 1000 s/mm^2^ was applied along with 30 diffusion gradient directions.

fMRI acquisition: fMRI was performed using spin echo - echo planner imaging sequence. The parameters are: TR = 2000 ms; TE = 15 ms; flip angle = 90°; number of repetitions: 200; slice thickness = 1 mm; matrix = 80 × 64; field of view = 25 × 25 mm.

### Data processing and analysis

#### DTI processing

DTI data were processed using the FMRIB software library (FSL) v6.0. The pre-processing steps were conducted as follows: (i) individual distortion correction to remove head motion and diminish the deformation. (ii) correction of gradient directions according to the corrected b vectors. (iii) brain extraction using bet function and generation of a brain mask according to b0 image. (iv) spatially normalized into the Waxholm space of the Sprague Dawley rat brain [43]. (v) calculation of DTI indices, including fractional anisotropy mean diffusivity, radial diffusivity, and axial diffusivity. Fractional anisotropy and mean diffusivity are typically regarded as indicators of superior white matter integrity [44]. Structurally intact white matter fibers are anticipated to exhibit higher fractional anisotropy values and lower mean diffusivity values [45]. Radial diffusivity reflects myelin content, with an increased radial diffusivity value signifying demyelination [46]. Axial diffusivity has been demonstrated to be sensitive to axonal injury, with elevated axial diffusivity values indicating axonal degeneration [13]. 4 ROIs with abundant myelinated axons including CCWM, AC, FN, and PC were extracted automatically based on atlas and assessed.

#### Resting state fMRI processing

The functional data were pre-processed and statistically analyzed using spmratIHEP [47, 48] based on statistical parametric mapping (SPM12) (Welcome Department of Imaging Science) software and DPARSF (http://rfmri.org/DPARSF) software. The main steps are: (i) magnification: the voxel size of the functional datasets of all rats was first multiplied by a factor of 5 to better approximate human dimensions. (ii) slice timing: the differences in acquisition times were corrected. (iii) realign: the temporal processed volumes were realigned to the first volume to remove the head motion. A mean image was generated over the 200 realigned volumes. No rat was excluded from the analysis because translation and rotation in x, y or z axis are less than 1 mm and 1°. (iv) spatial normalization: The re-aligned volumes were spatially standardized into the Paxinos & Watson space [49] according to their corresponding mean image. The normalized images were then resliced by 1.0 × 1.5 × 1.0 mm^3^ voxels. (v) smooth: a Gaussian kernel of 2  × 3  ×  2  mm^3^ Full Width at Half-maximum was used for the smooth of functional images. (vi) denoising: the smoothed series were 0.01–0.08 Hz band-pass filtered for the correction of head movement by regressing the translations and rotation of the head.

The functional connectivity was determined at ROI-level using correlational analyses. In detail, the averaged time courses from all voxels within ROIs were calculated as reference time courses. The strength of the FC was quantified using Pearson’s cross-correlation, which calculated the correlation coefficients between reference time course and individual time course. After z-transformation, DMN (left and right orbital frontal cortices, left and right prelimbic cortices, left and right frontal cortices, left and right anterior cingulate cortices, left and right hippocampus, left and right retrosplenial cortices and left and right temporal association cortices), and LCN (left and right motor cortices, left and right sensory cortices and left and right insula cortices) were extracted in line with previous studies [27, 50] for local network analysis between groups (Table S2). Benjamini-Hochberg false discovery rate (FDR) was applied for multiple comparison correction for the analysis of functional connectivity.

### Tissue preparation

Rats were injected with an overdose of sodium pentobarbital (100 mg/kg) and decapitated. For immunofluorescent staining, 12 rats (6 rats for LFB staining and 6 rats for immunofluorescence) per group were anesthetized and transcardially perfused with 0.9% NaCl followed by 4% paraformaldehyde. Brains were fixed in 4% paraformaldehyde overnight and dehydrated with 20% and 30% sucrose in PBS at 4 ℃. Then the brains were embedded in optimal cutting temperature compound and were sectioned coronally at the level of the corpus callosum using a freezing microtome (Leica, GER) to a thickness of 10 μm. For Western blot (6 rats per group) and ELISA (6 rats per group), white matter tissues were rapidly separated on the ice and were stored at −80℃.

### LFB staining

Brain sections were washed with 0.1 M PBS and incubated in 0.1% LFB solution (Solarbio, CN) at 56 ℃ for 8–10 h. After washing with 95% ethanol, sections were differentiated by 0.05% lithium carbonate solution and 70% enthanol, and dehydrated with graded ethanol and xylene. Images of the corpus callosum were captured by optical microscope (Olympus, BX43, JPN).

### Immunofluorescence

Brain samples were blocked with 10% bovine serum albumin, permeabilized with 0.3% Triton X-100, and incubated in primary antibodies to myelin basic protein (MBP, rabbit anti-MBP, 1:100, Proteintech, CN), myelin associated glycoprotein (MAG, rabbit anti-MAG, 1:100, Proteintech, CN), neurofilament-L (NF-L, rabbit anti-NF-L, 1:1000, Proteintech, CN), NF-M (rabbit anti-NF-M, 1:1000, Proteintech, CN), NF-H (rabbit anti-NF-H, 1:1000, Proteintech, CN), ionized calcium binding adapter molecule1 (Iba-1, goat anti-Iba-1, 1:500, Abcam, US), glial fibrillary acidic protein (GFAP, rabbit anti-GFAP, 1:1000, Abcam, US), iNOS (rabbit anti-iNOS, 1:1000, Proteintech, CN) and CD206 (mouse anti-CD206, 1:20000, Proteintech, CN) at 4 °C overnight. Subsequently, the sections were incubated using secondary antibodies from donkey anti-goat conjugated with Alexa Fluor 488 (1:1000, Invitrogen, US) or donkey anti-rabbit conjugated with Alexa Fluor 555 (1:1000, Invitrogen, US). Brain sections were captured using a fluorescent microscope (Olympus, BX43, JPN). Cells were counted by Image J (magnification, 200×) in corpus callosum (bregma: 1.56) according to Paxinos and Watson. All the other measurements were analyzed using ImageJ software.

### Western blot

White matter tissues were lysed with RIPA buffer solution (Thermo, US), supplemented with phosphatase inhibitors and protease inhibitor (Thermo, US). Bovine serum albumin (Thermo, US) was used for the measurement of protein concentration. Proteins (40 µg) were loaded onto a 10% SDS-PAGE gel for electrophoresis and transblotted to polyvinylidene difluoride membrane (Merck Millipore, US). The membranes were blocked with 5% non-fat milk in TBST, and incubated overnight at 4 ℃ with primary antibodies including antibodies against myelin basic protein (MBP, rabbit anti-MBP, 1:2000, Proteintech, CN), myelin associated glycoprotein (MAG, rabbit anti-MAG, 1:2000, Proteintech, CN), neurofilament-L (NF-L, rabbit anti-NF-L, 1:2000, Proteintech, CN), NF-M (rabbit anti-NF-M, 1:1000, Proteintech, CN), NF-H (rabbit anti-NF-H, 1:500, Proteintech, CN), iNOS (rabbit anti-iNOS, 1:2000, Proteintech, CN), TNF-α (rabbit anti-TNF-α, 1:500, Abcam, US) CD206 (mouse anti-CD206, 1:1000, Proteintech, CN), Arginase-1 (rabbit anti-Arginase-1, 1:5000, Proteintech, CN), gp91 phox (rabbit anti-gp91 phox, 1:5000, Abcam, US), gp47 phox (rabbit anti-gp47 phox, 1:200, Santa Cruz Biotechnology, US), gp67 phox (rabbit anti-gp67 phox, 1:100, Santa Cruz Biotechnology, US), GAPDH (rabbit anti- GAPDH, 1:2000, Abcam, US) and β-actin (rabbit anti-β-actin, 1:1000, Biodragon, CN). Then the membranes were incubated with IRDye 800CW anti-mouse IgG H&L (1:5000, Abcam, US) or Dylight 800 conjugate anti-rabbit (H + L) second antibody (1:5000, KPL, US). The images were recorded with an imaging system (Bio-Rad, US) and analyzed using Image J software.

### Stereotaxic surgeries and injections

After two weeks of treatment, 6 rats per group were anesthetized with isoflurane (5% for induction, 1.5–2.0% for maintenance) in a stereotaxic frame. To retrogradely trace the corpus callosum-anterior cingulate cortex long projection, cholera toxin subunit B (300 nl, CTB-488, Braincase, Wuhan, China) was injected into the left anterior cingulate cortex (AP 1.2 mm; L 0.5 mm; DV −2.7 mm) at a rate of 30 nl/min. Following a 3-day survival period, animals were fixation-perfused and tissue was collected. The native fluorescent signal was amplified using a cocktail of mouse anti-CTB (1:500, Abcam, Catalog number: ab35988) followed by a cocktail of donkey anti-mouse IgG AF488 secondary antibody (1:400, Jackson Immunoresearch, RRID: AB_ 2341099). The number of CTB + cells in the right anterior cingulate cortex and the area of positive pixels in the corpus callosum were detected by immunofluorescence imaging with a microscope.

### Statistical analysis

Statistical analysis was conducted using GraphPad Prism 8 (San Diego, CA, US). A paired t-test was used to compare DTI indices which were recorded twice at 1 and 2 weeks after BCCAO operation. Results of brain networks, LFB, immunofluorescence and Western Blot were compared between groups via one-way ANOVA followed by Tukey’s *post hoc* test. Novel object recognition test and eight-arm maze data were analyzed using two-way repeated measures ANOVA. A *p*-value of less than 0.05 was considered statistically significant. Non-normally distributed data were analyzed using Kruskal-Wallis and Dunn’s *post hoc* test. Descriptive statistics are expressed as mean ± standard deviations (SD).

Correlation analysis was performed with Pearson’s correlation. Mediation analysis was performed with PROCESS v 3.4 (https://processmacro.org/index.html). 5000 permutation was set for bootstrapping. Direct effects, indirect effects, and the percentages of mediator effects accounting for the total effects were calculated and reported.

## Supplementary Information

Below is the link to the electronic supplementary material.

Supplementary Material 1.

## Data Availability

The data used to support the findings of this study are available from the corresponding authors upon request.

## Abbreviations

AC: Anterior commissure
BCCAO: Bilateral common carotid artery occlusion
CCWM: Corpus callosum and associated subcortical white matter
DTI: Diffusion tensor imaging
DMN: Default mode network
FN: Fornix
LCN: Lateral cortical network
LFB: Luxol fast blue
PC: Posterior commissure
ROIs: Regions of interest
VCID: Vascular cognitive impairment and dementia
WMA: White matter abnormalities
