# Supplementary material for: Rewiring brain structural and functional disconnection with acupuncture in rat model of vascular cognitive impairment and dementia
Source: Biol Res. 2026 Jan 19;59:10. doi: 10.1186/s40659-026-00670-5 (PMC12903555; doi:10.1186/s40659-026-00670-5)
Supplement: Supplementary file 1 — Supplementary Material 1. [file 40659_2026_670_MOESM1_ESM.docx]

**Supplementary information**

**Rewiring brain structural and functional disconnection with acupuncture in rat model of vascular cognitive impairment and dementia**


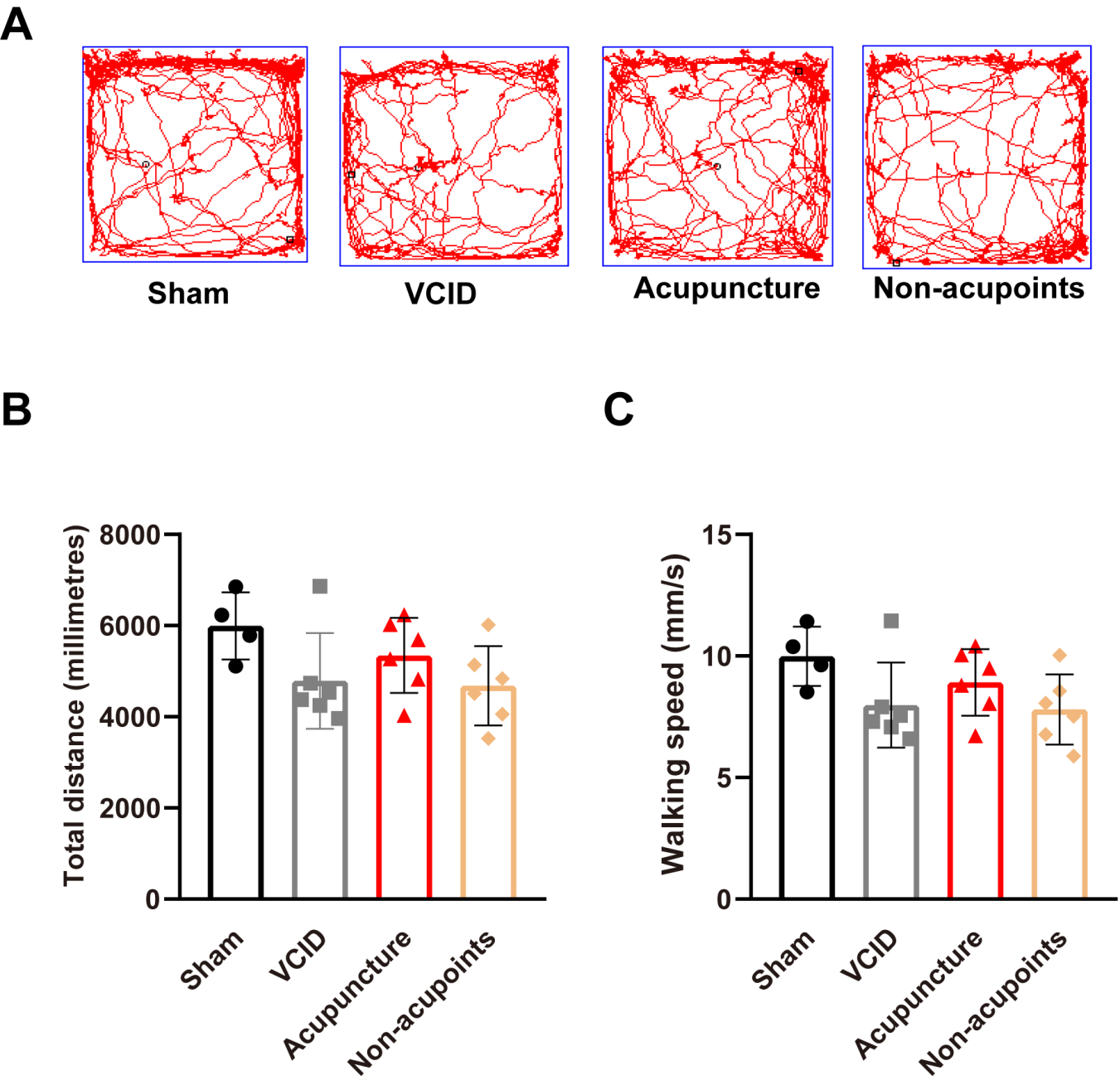


**Fig. S1. Measurements for locomotor activity were performed by open field test.** (A) Representative walking traces of each group of rats. (B) Total distance. (C) Walking speed. One-way ANOVA with Tukey’s post-hoc test. Data are presented as mean ± SD (n=6 per group).


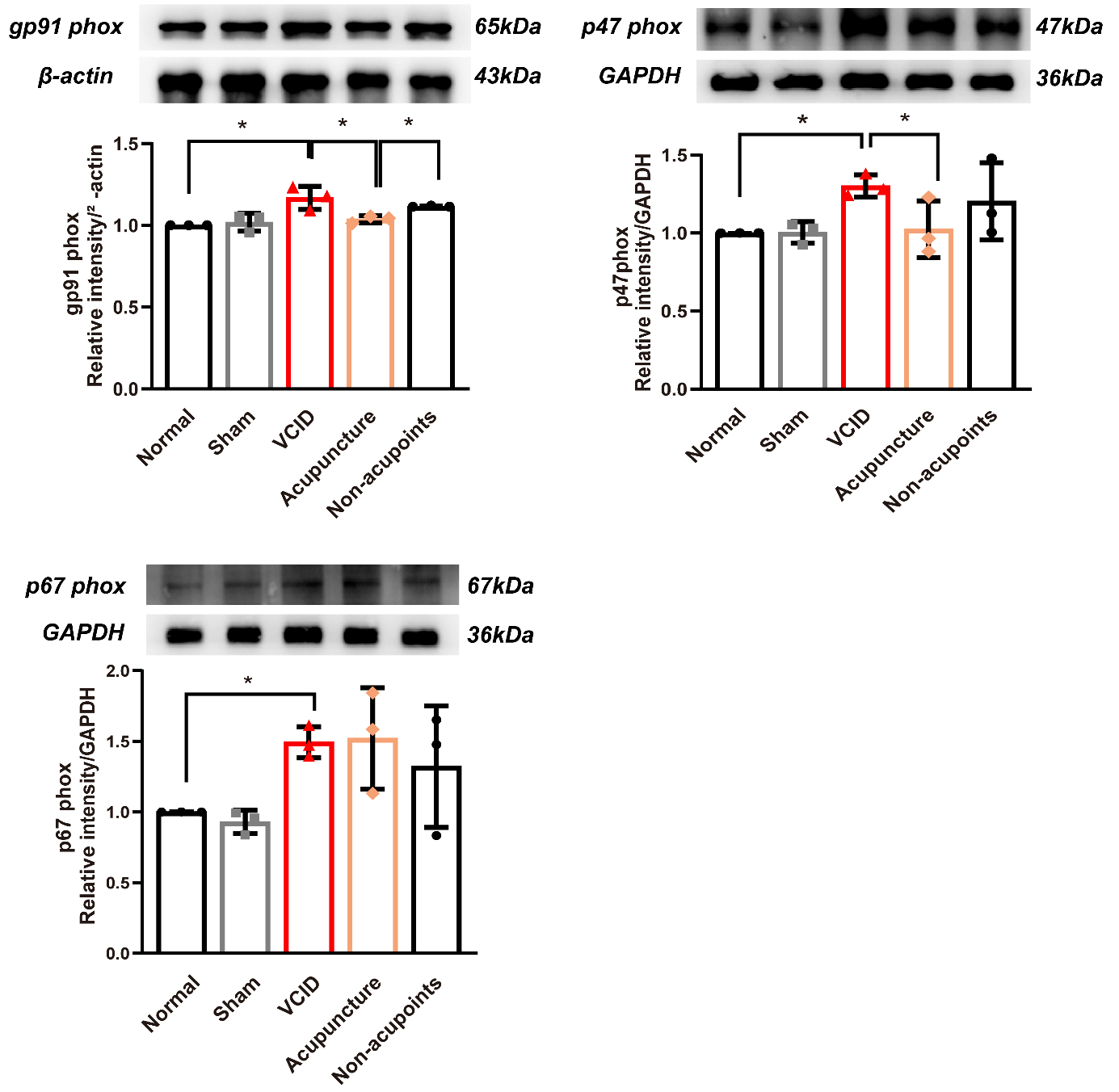


**Fig. S2 Acupuncture regulated part of oxidative stress marker genes NADPH oxidase subunits expression in hippocampus of VCID rats.** The protein level of p47phox, p67phox, gp91phox and its corresponding GAPDH or β -actin bands were determined using Western blot analysis. The following histogram shows the quantitative densitometry of the blots in each group. Data are presented as mean ± SD (n=3 per group). **p*＜0.05. Normal: Normal group, Sham: sham-operated group, VCID: BCCAO-operated group, Acupuncture: VCID + acupuncture at GV20 and ST36 group, Non-acupoints: VCID + acupuncture at non-acupoints group.

**Fig. S3 Comparisons of DTI indices between groups 14 days after BCCAO surgery.** Quantification of DTI indices in representative white matter regions using diffusion tensor imaging. Data are presented as mean ± SEM (n = 8 per group). *p＜0.05, ** p＜0.01, *** p＜0.001, **** p＜0.0001, respectively. FA: fractional anisotropy, MD: mean diffusivity, RD: radial diffusivity, AD: axial diffusivity, CCWM: corpus callosum and associated subcortical white matter, AC: anterior commissure, FN: fornix, PC: posterior commissure, Sham: sham-operated group, VCID: BCCAO-operated group, Acupuncture: VCID + acupuncture at GV20 and ST36 group, Non-acupoints: VCID + acupuncture at non-acupoints group.


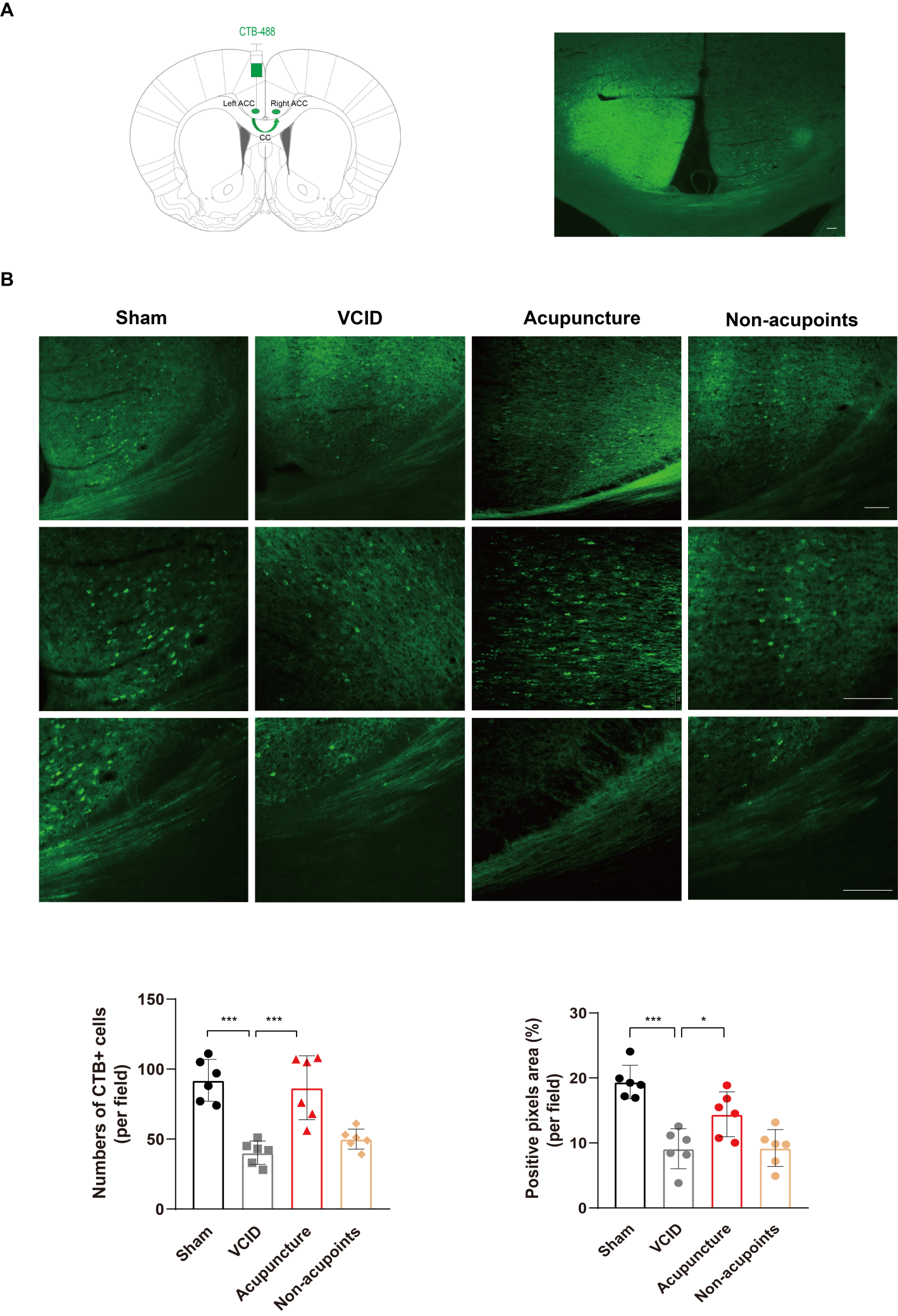


**Fig. S4 Acupuncture improved corpus callosum-anterior cingulate cortex axis.** (A) CTB-488 is injected into left anterior cingulate cortex. Image shows CTB-488 expressed in left anterior cingulate cortex, transited through the corpus callosum and retrograde to right anterior cingulate cortex, Scale bar: 100μm. (B) Representative images demonstrate the immunofluorescent labeling of CTB+ cells in the right anterior cingulate cortex and positive pixels area in the corpus callosum, Scale bar: 100μm. One-way ANOVA with Tukey’s post-hoc test. Data are presented as mean ± SD (n = 6 per group). * *p*＜0.05 and *** *p*＜0.001, respectively. ACC: anterior cingulate cortex, CC: corpus callosum.


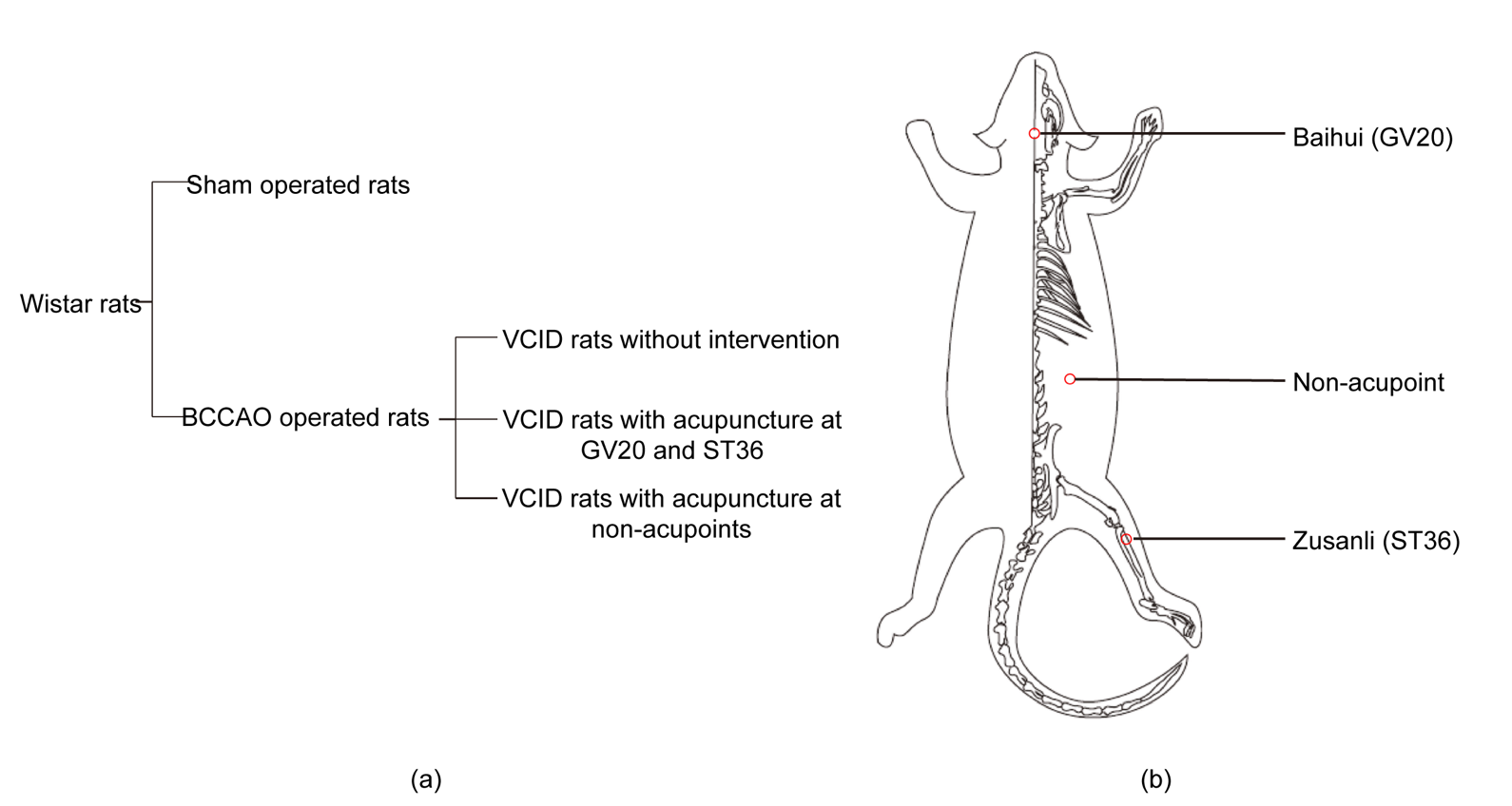


**Fig. S5. Animal groups and acupoints location.** GV20: Baihui acupoint, ST36: Zusanli acupoint, BCCAO: bilateral common carotid artery occlusion, VCID: rats with BCCAO suegery.

**Table S1. Rats body weight before and during eight-arm radial maze training**

| Groups | Pre | Day1 | Day2 | Day3 | Day4 | Day5 | Day6 | Day7 | Day8 | Day9 |
| --- | --- | --- | --- | --- | --- | --- | --- | --- | --- | --- |
| Sham1 | 318 | 290 | 283 | 304 | 280 | 283 | 286 | 295 | 283 | 298 |
| Sham2 | 315 | 300 | 293 | 316 | 306 | 314 | 320 | 313 | 309 | 309 |
| Sham3 | 307 | 289 | 286 | 312 | 291 | 288 | 292 | 288 | 285 | 286 |
| Sham4 | 302 | 250 | 240 | 294 | 246 | 246 | 250 | 249 | 239 | 246 |
| Sham5 | 312 | 286 | 285 | 322 | 296 | 290 | 296 | 301 | 299 | 302 |
| Sham6 | 304 | 267 | 266 | 306 | 285 | 287 | 296 | 293 | 287 | 292 |
| Sham7 | 286 | 262 | 260 | 263 | 260 | 258 | 267 | 267 | 262 | 270 |
| Sham8 | 296 | 272 | 258 | 262 | 255 | 253 | 263 | 270 | 265 | 273 |
| VCID1 | 322 | 296 | 281 | 288 | 291 | 302 | 308 | 330 | 326 | 326 |
| VCID2 | 301 | 250 | 242 | 259 | 248 | 257 | 260 | 279 | 278 | 278 |
| VCID3 | 305 | 266 | 256 | 260 | 279 | 280 | 284 | 306 | 297 | 307 |
| VCID4 | 298 | 242 | 233 | 236 | 239 | 238 | 241 | 251 | 253 | 258 |
| VCID5 | 310 | 278 | 253 | 247 | 244 | 254 | 260 | 278 | 255 | 253 |
| VCID6 | 313 | 254 | 236 | 246 | 260 | 259 | 267 | 276 | 278 | 273 |
| VCID7 | 307 | 282 | 266 | 271 | 263 | 260 | 264 | 268 | 268 | 274 |
| VCID8 | 302 | 268 | 258 | 265 | 265 | 260 | 265 | 279 | 278 | 278 |
| Acu1 | 308 | 275 | 266 | 273 | 265 | 258 | 267 | 280 | 275 | 285 |
| Acu2 | 311 | 260 | 250 | 256 | 249 | 258 | 250 | 269 | 254 | 253 |
| Acu3 | 282 | 221 | 218 | 276 | 258 | 240 | 223 | 241 | 234 | 232 |
| Acu4 | 298 | 258 | 248 | 280 | 252 | 265 | 275 | 290 | 276 | 273 |
| Acu5 | 289 | 275 | 260 | 280 | 264 | 268 | 276 | 294 | 275 | 272 |
| Acu6 | 278 | 256 | 257 | 259 | 238 | 248 | 246 | 251 | 244 | 239 |
| Acu7 | 294 | 269 | 282 | 289 | 266 | 277 | 266 | 286 | 280 | 278 |
| Acu8 | 278 | 275 | 278 | 281 | 269 | 275 | 274 | 285 | 275 | 267 |
| Non-acu1 | 296 | 275 | 263 | 278 | 267 | 272 | 275 | 288 | 270 | 276 |
| Non-acu2 | 280 | 270 | 250 | 290 | 280 | 276 | 276 | 283 | 292 | 294 |
| Non-acu3 | 283 | 265 | 263 | 286 | 278 | 292 | 303 | 326 | 318 | 306 |
| Non-acu4 | 306 | 290 | 273 | 280 | 273 | 282 | 278 | 305 | 306 | 310 |
| Non-acu5 | 269 | 296 | 296 | 273 | 252 | 256 | 267 | 288 | 269 | 276 |
| Non-acu6 | 266 | 246 | 243 | 263 | 242 | 255 | 260 | 281 | 269 | 274 |
| Non-acu7 | 262 | 231 | 238 | 262 | 246 | 250 | 258 | 278 | 275 | 270 |
| Non-acu8 | 257 | 237 | 238 | 243 | 230 | 237 | 228 | 225 | 217 | 222 |

**Table S2. Anatomical labels of each ROI in functional MRI process**

| ROIs | Coordinates (mm) | | |
| --- | --- | --- | --- |
|  | X | Y | Z |
| Left anterior cingulate cortex | -3.9 | 15.5 | 29.4 |
| Right anterior cingulate cortex | 3.9 | 14.5 | 29.4 |
| Left frontal cortex | -33.9 | 15.5 | 17.4 |
| Right frontal cortex | 28.1 | 16.5 | 17.4 |
| Left hippocampus | -31.9 | -30.5 | 21.4 |
| Right hippocampus | 31.1 | -29.5 | 21.4 |
| Left orbital cortex | -10.9 | 19.5 | 13.4 |
| Right orbital cortex | 10.9 | 20.5 | 13.4 |
| Left retrosplenial cortex | -3.1 | -16.5 | 37.4 |
| Right retrosplenial cortex | 3.1 | -13.5 | 37.4 |
| Left prelimbic cortex | -4.9 | 19.5 | 20.4 |
| Right prelimbic cortex | 2.1 | 19.5 | 20.4 |
| Left temporal association cortex | -50.9 | -30.5 | 23.4 |
| Right temporal association cortex | 50.1 | -31.5 | 23.4 |
| Left insula cortex | -44.9 | 1.5 | 1.4 |
| Right insula cortex | 43.1 | 1.5 | 1.4 |
| Left motor cortex | -13.1 | -4.5 | 34.4 |
| Right motor cortex | 13.1 | -5.5 | 34.4 |
| Left sensory cortex | -43.1 | -2.5 | 26.4 |
| Right sensory sortex | 43.1 | -3.5 | 26.4 |

**Table S3 A *post hoc* correlation analysis between the white matter integrity of corpus callosum and revisiting error**

|  | White matter integrity | *P* value | Correlation with working memory |
| --- | --- | --- | --- |
|  |  |  | r |
| Corpus callosum | Fractional anisotropy | **＜0.0001** | 0.792 |
|  | Mean diffusivity | **0.003** | 0.510 |
|  | Radial diffusivity | 0.114 | 0.285 |
|  | Axial diffusivity | **0.009** | 0.454 |

**Table S4 A *post hoc* correlation analysis between the altered functional connectivity and revisiting error**

|  | Functional connectivity | *P* value | Correlation with working memory |
| --- | --- | --- | --- |
|  |  |  | r |
| Intra-hemisphere | Left orbital cortex-left hippocampus | **0.001** | 0.555 |
|  | Right hippocampus-right anterior cingulate cortex | **0.027** | 0.392 |
| Inter-hemisphere | Left hippocampus-right orbital cortex | **＜0.0001** | 0.66 |
|  | Left hippocampus-right hippocampus | 0.292 | 0.192 |

**Table S5. The location of the acupoints**

| Acupoints | Anatomical location |
| --- | --- |
| Baihui (GV20) | Midline of the head, approximately midway on the line connecting the apices of the auricles. |
| Zusanli (ST36) | 2 mm lateral to the anterior tubercle of the tibia, and 5 mm below the capitulum fibulae under the knee joint |
| Nonacupoint | 1cm left side next to GV20 or 1cm outside next to ST36 |
